# A Comparative Analysis of Mechanical Properties of Polyetheretherketone (PEEK) vs. Standard Materials Used in Orthodontic Fixed Appliances: A Systematic Review

**DOI:** 10.3390/polym16091271

**Published:** 2024-05-02

**Authors:** Pyi Phyo Win, Oak Gar Moe, Daniel De-Shing Chen, Tzu-Yu Peng, Johnson Hsin-Chung Cheng

**Affiliations:** 1 School of Dentistry, College of Oral Medicine, Taipei Medical University, Taipei 11031, Taiwan; d204112004@tmu.edu.tw (P.P.W.); star_align@tmu.edu.tw (D.D.-S.C.); 2 Division of Orthodontics, Department of Dentistry, Taipei Medical University Hospital, Taipei 11031, Taiwan; 3 School of Dentistry, University of Michigan, Ann Arbor, MI 48104, USA; oakgar@umich.edu

**Keywords:** polyetheretherketone, PEEK, polymer, dentistry, orthodontics, fixed appliances, mechanical properties

## Abstract

Polyetheretherketone (PEEK), an organic thermoplastic polymer, has gained interest in dentistry due to its excellent mechanical strength, flexibility, and biocompatibility. Furthermore, the ability to utilize CAD/CAM in the fabrication of PEEK enhances accuracy, reliability, and efficiency while also saving time. Hence, several orthodontic studies have explored the utilization of PEEK in various applications, such as archwires, brackets, fixed lingual retainers, palatal expansion devices, transpalatal arches, Tübingen palatal plates, different types of space maintainers, mini-implant insertion guides, and more. However, a complete systematic review of the available data comparing the performance of PEEK with traditional orthodontic materials has not yet been conducted. Therefore, this systematic review seeks to assess if PEEK material meets the required mechanical criteria to serve as an alternative to conventional orthodontic appliances. To ensure clarity and precision, this review will specifically concentrate on fixed appliances. This systemic review followed the PRISMA guidelines and utilized databases including PubMed/MEDLINE, Embase, Springer, Web of Science, and Wiley. Searches were restricted to English language articles from January 2013 to February 2024. Keywords such as “Polyetheretherketone” or “PEEK” and “Orthodontic” or “Orthodontic device” or “Orthodontic materials” were employed across all databases. Nine studies were incorporated, covering orthodontic archwires, brackets, and fixed lingual retainers. Based on the reviewed literature, PEEK demonstrates promising potential in orthodontic fixed appliances, offering advantages in force delivery, friction reduction, and aesthetic appeal. Further research is needed to fully explore its capabilities and optimize its application in clinical practice.

## 1. Introduction

In our modern era, as the usage of social media continues to rise, there is a growing demand for visual esthetics and appeal. Moreover, one study has determined that a harmonious and attractive smile could enhance the chances of securing a job interview or being offered employment [1], a critical factor in today’s intensely competitive time. Hence, the alignment of teeth significantly influences the act of smiling, and opting for an orthodontic treatment has become one of the popular options. Furthermore, there are various alternative materials in orthodontic treatment. If a patient is concerned about the visibility of the brackets or they are allergic to metal, they have the option to choose non-metallic esthetic brackets which are fixed onto the teeth [2], or a clear aligner treatment which is removable [3]. However, due to its high dependence on patients’ compliance and some limitations of the clear aligner in tooth movement, it may not be suitable for all cases, and fixed appliances could become the preferred choice [4]. Esthetic brackets are commonly made of ceramic or plastic. One study has concluded that plastic brackets show more deformation than ceramic and stainless steel (SS) brackets under the same wire load [5]. Moreover, in order to reinforce durability, ceramic brackets are noticeably bulkier than metal ones which can affect the comfort of the patient [6]. In addition to esthetic brackets, tooth-colored archwires have also been introduced which can seamlessly blend with the brackets and the teeth, providing effective camouflage and enhancing the overall esthetic appearance [7]. However, in 2017, Mikuleuicz et al. stated that although commercially available esthetic archwires were visually pleasing, they still needed more improvements in mechanical properties such as rigidity, flexibility, and durability [8]. As technology undergoes significant advancement, the integration of digital workflow has become increasingly prevalent in orthodontic treatment. This results in increased accuracy, predictability, and the avoidance of complications [9]. For instance, one of the factors of a fixed lingual retainer that causes undesired tooth movement after orthodontic treatment is poor fabrication of the retainer wire which could exert forces onto the fixed teeth [10]. Therefore, several studies have tested the application of different types of computer-aided design and computer-aided manufacturing (CAD/CAM)-fabricated materials in orthodontic treatment for better clinical outcomes. Among those materials, polyetheretherketone (PEEK) stands out as a potential candidate due to its excellent mechanical and physical properties, as well as its biocompatibility [11]. In addition, PEEK demonstrates remarkable versatility, as it can be utilized not only in milling but also in 3D printing, despite its high melting point of 343 °C [12]. Therefore, researchers are increasingly drawn to explore its alternative applications in orthodontic treatment [13]. Nevertheless, a comprehensive systematic review of the existing data concerning the performance of PEEK over conventional orthodontic materials is still unavailable. Hence, the aim of this systematic review is to assess whether PEEK material possesses the necessary mechanical characteristics to be considered as an alternative to conventional orthodontic fixed appliances.

## 2. Materials and Methods

### 2.1. Eligibility Criteria

This systematic review was conducted following to the guidelines of the Preferred Reporting Items for Systematic Reviews and Meta-Analyses (PRISMA) [14].

- The inclusion criteria were defined according to PICOS framework:
- Sample (P) = PEEK materials;
- Intervention (I) = Applications in fixed orthodontic appliances;
- Comparison (C) = Mechanical properties of conventional orthodontic materials;
- Outcome (O) = Quantitative analysis of different kinds of mechanical investigation;
- Study design (S) = In vivo or in vitro experimental studies.

The exclusion criteria include articles that emphasized other polymers apart from PEEK and those that did not compare PEEK with conventional orthodontic materials. Additionally, studies that employed PEEK in removable orthodontic appliances were also excluded. Systematic reviews, meta-analysis, case reports, and book chapters were also omitted in this review.

### 2.2. Information Sources

Searching was carried out through five electronic databases including PubMed/MEDLINE, Embase, Springer, Web of Science, and Wiley. The search was confined to the English language exclusively, spanning from January 2013 to February 2024. Keywords such as “Polyetheretherketone” or “PEEK” and “Orthodontic” or “Orthodontic device” or “Orthodontic materials” were utilized across all databases. The resultant articles were exported to a reference manager (EndNote 20.6, Clarivate Analytics, Philadelphia, PA, USA) to streamline the following procedures: the identification and removal of duplicates and the elimination of articles that were not related to this systematic review. The reference lists of the included studies were also manually searched to identify any other articles that might be relevant.

### 2.3. Search Strategy and Study Selection

Two independent reviewers (P.P.W. and O.G.M.) screened the titles and the abstracts of the articles after removal of the duplicates. In the event of a difference in the number of selected articles, preference was given to the larger count. The final selection was performed by analyzing the complete text of each chosen article and if there was a disagreement, it was discussed with reviewers (T.-Y.P. and J.H.-C.C.).

### 2.4. Data Extraction

The two reviewers (P.P.W. and O.G.M.) dealt with the extraction of the following data: the authors, year of publication, the application of PEEK material, the different conventional orthodontic materials that were compared to PEEK, types of applied tests, and the conclusions. Later, the extracted data were checked by reviewers (T.-Y.P. and J.H.-C.C.).

### 2.5. Assessment of Risk of Bias in Individual Studies

Since all the selected studies were experimental in vitro studies, the Quin tool was used to access the risk of bias [15]. There were 12 parameters to be considered in total. Each category scored “two points” when adequately specified, “one point” if inadequately specified, and “zero points” if not specified. Moreover, if a specific category was not applicable in one study, it was excluded from the calculation. The final score was calculated according to the following formula:

$$\mathrm{Final}\;\mathrm{score}=\frac{\mathrm{Total}\;\mathrm{score}\;\text{×}\;100}{2\;\text{×}\;\mathrm{number}\;\mathrm{of}\;\mathrm{criteria}\;\mathrm{applicable}}$$

According to the resulting final score, each study was graded as high, medium, or low risk where more than 70% was stated as low risk of bias, between 50% and 70% was claimed as medium risk of bias, and less than 50% was said to be high risk of bias. Two independent reviewers (P.P.W. and O.G.M.) performed this risk of bias assessment individually and any disagreement was discussed with reviewers (D.D.-S.C. and J.H.-C.C.) if necessary.

### 2.6. Summary of Measurements and Synthesis of Results

The measurements for the tests in each study were Von-Mises stress (MPa), applied forces (N), amount of deformation (mm), and coefficient of friction (μ). Therefore, the outcomes were continuous in nature where mean values and standard deviations of the results were selected for comparison between PEEK and the control.

Regarding the methodology, there was a large diversity between the studies, for instance, various applications, different dimensions of specimens, and study designs and, therefore, a meta-analysis was not possible.

## 3. Results

### 3.1. Study Selection

**Figure 1:**
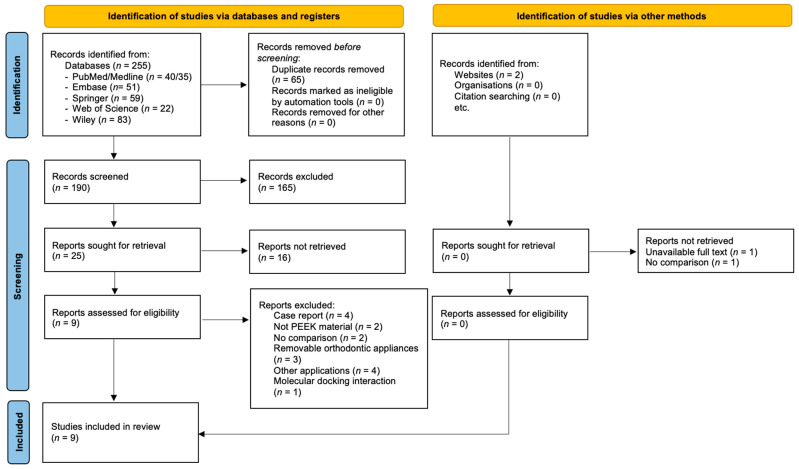
PRISMA flow diagram showing the literature selection procedure.

A flow diagram of the literature selection procedure as outlined in PRISMA is shown in Figure 1. The database searches identified 255 references: PubMed/MEDLINE (*n* = 40/35), Embase (*n* = 51), Springer (*n* = 59), Web of Science (*n* = 22), and Wiley (*n* = 83). After duplicate removal, 190 studies remained. Hand searching revealed two articles where one did not include any comparison with a conventional retainer wire and, for another paper, the full text was unavailable. The titles and abstracts were screened, and 165 articles were discarded. Twenty-five articles from the database were selected for full-text evaluation and applying the eligibility criteria. Of these, 16 articles were excluded for reasons such as using different materials other than PEEK (*n* = 2), using PEEK in removable appliances (*n* = 3), comparing the results between different alterations of PEEK without including the conventional material (*n* = 2), investigating the molecular docking interaction of PEEK used in orthodontic mini-implant fabrication (*n* = 1), examining PEEK in a case report (*n* = 4), and 4 other articles investigated PEEK in other dental fields such as alveolar bone augmentation (*n* = 1), denture components (*n* = 2), and insertion guides for purely mini-implant-borne rapid maxillary expanders (*n* = 1). As a result, nine articles were selected for this systematic review.

### 3.2. Study Characteristics 

All the studies were in vitro experimental studies (Table 1). 

Among the selected nine articles, three articles were regarding orthodontic wires where two of them studied PEEK used in actual orthodontic archwires [16,17] while another one identified the advantages of using PEEK as a sleeve covering the wire [18]. The former two articles focused mainly on determining the mechanical strength of the wires by using a three-point bending test and a bending creep test or stress relaxation test. However, Maekawa et al. [16] did not compare PEEK with other conventional orthodontic materials in the bending creep test. Furthermore, different dimensions of PEEK archwires were used during their experiments where Maekawa et al. [16] utilized “0.039 × 0.039 in^2^” (≈1.0 × 1.0 mm^2^) PEEK wires while Tada et al. [17] investigated the cross-sectional dimensions of “0.016 × 0.022 in^2^”, “0.019 × 0.025 in^2^”, and “0.016 in” PEEK wires. Moreover, Tada et al. [17] considered three types of wire ligation methods in a “0.022 × 0.028 in^2^” bracket slot—no ligation (NL), elastomeric ligation (EL), and slot lid ligation (SL) conditions—during the bending test and further measured the friction between the PEEK wires and the orthodontic brackets. Shirakawa et al. [18] proposed the idea of using PEEK as an archwire sleeve. The study mainly covered important factors in the sleeve covering scenarios such as friction development and amount of bracket surface roughness. In this experiment, two types of PEEK tubes which could accommodate the sizes of “0.018 in” and “0.0215 × 0.028 in^2^” wires were prepared. According to the results of the experiment, the difference between using a PEEK cover and not using it on the conventional orthodontic wires was evaluated. 

One study was concerned with using PEEK instead of ceramic as an esthetic orthodontic bracket [19]. In this article, the surface characteristics, amount of sliding friction, abrasive wear, elastic modulus, and hardness of the PEEK brackets were evaluated compared to the conventional ceramic materials. PEEK disks and ceramic disks, each with a diameter of “0.197 in” (≈5 mm) and thickness of “0.079 in” (≈2 mm), were used for surface roughness and morphology examination. The surface roughness and morphology of the specimens were assessed using a laser profilometer device. Due to the inconsistent compatibility of the base and the slot of PEEK brackets with those of commercially available ceramic brackets, flat disks were used instead of actual brackets. 

Another five articles were related to orthodontic fixed lingual retainers. Three of them assessed the bonding strength of PEEK material [20,21,22]. Among them, two articles [20,21] conducted a debonding procedure after the aging process to evaluate long-term survival rate. Furthermore, in addition to the aging procedure, Alabbadi et al. inserted a 1mm hand-drilled hole at the center of a PEEK retention pad to reinforce the adhesion strength [20]. Kadhum et al. performed varying surface treatments on the PEEK retainer specimens to determine the most efficient surface treatment approach [22]. In that article, the grouping of PEEK retainer wires comprised three categories: those with no surface treatment, those subjected to air abrasion, and those air-abraded followed by conditioning with visio.link. Additionally, the outcomes of all the above tested specimens were compared with commonly used orthodontic fixed lingual retainers [20,21,22]. Nevertheless, the dimensions of the PEEK retainers were customized based on their individual preferences which led to inherent variations. Certain individuals favored fabricating the PEEK retainers with large surface areas [20,21] while others chose to follow the dimensions of actual wires [22]. Hence, Win et al. carried out an experiment to find out the optimal dimension of the PEEK-made fixed lingual retainer by using the finite element method and a laboratory three-point bending test and, finally, determine the best dimension of the retainer by comparing their results with the traditional SS wire [23]. However, the article did not include consideration regarding bonding strength and solely focused on mechanical strength. One of the key requirements of a fixed lingual retainer is the ability to hold the teeth in a particular alignment without restricting the physiologic tooth movement. Therefore, Roser et al., who previously tested the bonding strength of a PEEK retainer, later examined the stiffness of a “0.047 × 0.138 in^2^” (≈1.2 × 3.5 mm^2^) PEEK retainer by evaluating its influence on vertical and horizontal tooth mobility under the universal testing machine [24]. The test results were also compared with a conventional five-stranded multistranded retainer.

### 3.3. Risk of Bias in Studies

Table 2 presents a comprehensive bias assessment of the selected articles, indicating that all the reviewed studies exhibited a medium risk of bias except two articles showing a low risk of bias. Notably, common concerns identified across these studies included issues related to “sample size calculation”, “operator detail”, “outcome assessor”, and “blinding”. None of the articles provided information regarding the blinding procedures during the specimen testing phase. Moreover, one study was categorized as inadequately specified due to its broad demonstration of aims and objectives, as well as its lack of detailed explanation of the statistical test employed [16]. Significantly, Roser et al. reported that all procedures were conducted by a dentist, addressing the “operator detail” and “outcome assessor” aspects [21], while Alabbadi et al. provided details about the sample size calculation [20]. However, the former article solely listed the material types without describing the dimensions of the test samples and the comparison group [21]. Later, in a subsequent experiment conducted by the same authors, Roser et al., the dimensions of the materials were clearly provided [24].

### 3.4. Results of Individual Studies

While conducting the search, PEEK was investigated in the context of orthodontic wires, archwire sleeves, orthodontic brackets, and fixed lingual retainers. Furthermore, the outcomes were compared with those of conventional orthodontic materials. 

In 2015, Maekawa et al. [16] demonstrated that the flexural loads of PEEK wires were similar to those of Ni-Ti wires but lower when compared to SS and Co-Cr wires. Alternatively, PEEK exhibited only 0.2 mm of displacement after being deflected to 2.0 mm whereas SS and Co-Cr wires were permanently deflected to around 1.0 mm. Ni-Ti wires did not show any permanent deformation. 

In 2017, Tada et al. [17] performed a three-point bending test under three conditions: NL, EL, and SL. After the test, the results showed that the bending load of Ni-Ti wires was larger than that of three types of PEEK wires when using NL and EL conditions. However, “0.019 × 0.025 in^2^” in the SL method preserved a greater deflection load than the Ni-Ti wires in term of loading (2mm) and unloading (1.5mm) conditions. Furthermore, a similar level of permanent deformation to the Ni-Ti wire was observed when the PEEK wires were fixed by SL. Moreover, a stress relaxation test for 24 hrs revealed that stress reduction rates of “0.016 × 0.022 in^2^” and “0.019 × 0.025 in^2^” PEEK wires were lower than those of Ni-Ti wires. The amount of friction generated between the PEEK wires and brackets resembled that of Ni-Ti wires and there was no significance between them. However, scanning electron microscope (SEM) images of a “0.019 × 0.025 in^2^” PEEK wire displayed a smoother surface than that of a Ni-Ti wire after the friction test. 

In 2018, Shirakawa et al. [18] introduced an alternative approach in which a PEEK tube is designed to encase the orthodontic archwire, rather than the fabrication of actual PEEK orthodontic wires. A friction test using a PEEK cover and not using it was performed and the results were compared. The surface roughness of the bracket slots after the friction test was also measured. Friction values were noticeably lower in all types of wire when protected by the PEEK cover, except for the “0.016 in” Ni-Ti wire, where there was no significant difference between using and not using PEEK. Regarding the surface of the brackets, those equipped with PEEK covers remained intact whereas those without PEEK exhibited several scratches due to direct wire-to-bracket contact.

In 2024, Wu et al. [19] investigated the use of PEEK as an esthetic orthodontic bracket by comparing it with standard ceramic material. Regarding the surface analysis, there was no significant difference between the two groups. Remarkably, the outcomes of the friction test revealed that PEEK exhibited a lower friction value (0.157 ± 0.050 µm) compared to ceramic (0.297 ± 0.062 µm) and this difference was statistically significant. After examination of the tested samples under SEM, ceramic expressed an abrasive wear style with chipping debris while the PEEK surface still maintained a smooth appearance without any noticeable debris, suggesting an adhesive wear pattern. The nano-dentation test indicated that ceramic was harder and less flexible than PEEK materials. 

In the context of using PEEK as an orthodontic fixed lingual retainer, positive data on PEEK materials were recorded by Kadhum et al. [22] and Win et al. [23], while Alabbadi et al. [20] and two articles by Roser et al. [21,24] showed unfavorable outcomes for PEEK as an alternative fixed lingual retainer. 

According to Kadhum et al. [22], they tested a “0.031 in” (≈0.8 mm) round PEEK retainer wire and observed comparable performance regardless of surface treatment when compared to conventional metallic wires. After evaluating the debonding test from acrylic blocks and bovine teeth, there was no statistically significant difference between the groups, except for the dead-soft coaxial wire. However, although the above metallic wire produces a higher ultimate failure strength, this often results in a significant amount of permanent deflection which has higher risk of unwanted tooth movement. The pattern of failure of PEEK wires, especially air-abraded specimens, and those both air-abraded and treated with visio.link (Bredent Medical GmbH & Co. KG, Senden, Germany) specimens were wire-ruptured with a minor composite fracture. Interestingly, both PEEK groups demonstrated a greater pull-out force compared to the non-surface treatment group and other conventional metallic retainers. 

In 2023, the mechanical strength of PEEK as a fixed orthodontic retainer was solely tested by Win et al. [23]. It was found that a “0.036 in” SS retainer was significantly stronger than a PEEK wire. However, in general, being strong enough to withstand the applied pressure (tongue pressure ≈ 15 N) is preferable because excessive strength can lead to a significant amount of deformation. PEEK with a hemi-elliptical, cross-sectional shape, having a thickness of “0.039 in” (≈1.0 mm) and width of “0.059 in” (≈1.5 mm) showed sufficient strength to withstand the force during a tongue pressure test. Hence, the permanent distortion of PEEK wires was significantly lower than that of SS wires. Both SS and PEEK were not strong enough to withstand the biting force. 

Alabbadi et al. [20] performed a debonding failure test between PEEK and a braided lingual retainer wire. All the test samples survived the aging procedure before the debonding failure test. After the examination, the SS retainer wire showed a significantly higher debonding force (45.73 ± 4.48 N) than the PEEK retainer (86.81± 4.59 N). Moreover, a higher connector displacement occurred in the conventional retainer (1.33 ± 0.30 mm) when compared to the PEEK wire (0.13 ± 0.07 mm). In contrast, adhesive remanent index (ARI) score was significantly lower in the metal wire (2.33 ± 0.35) than the PEEK retainer (3.00 ± 0.00).

Roser et al. conducted an aging and stability test for a PEEK-made fixed lingual retainer [21]. According to their results, conventional twistflex retainers were still the gold standard. On the other hand, all eight PEEK retainer samples failed to survive the aging process and did not even get the chance to undergo the fracture resistance bending test. 

In addition, the same as the previous author, Roser et al. also examined the restriction of tooth mobility affected by the PEEK retainer [24]. It restricted more horizontal tooth mobility (73%) than a conventional multistranded retainer (44%). However, a similar restriction of vertical tooth mobility was found between PEEK (21%) and multistranded (22%) retainers. These results show that the design of commercially accessible PEEK retainers significantly restricted tooth mobility more than the multistranded retainers, which could harm the periodontal ligament and less flexibility could increase stress on the bonding system.

## 4. Discussion

This systematic review evaluated the existing literature on the use of PEEK as an alternative material in fixed orthodontic appliances. The articles that carried out experiments through comparison with traditional orthodontic materials were selected for this review. This approach clearly demonstrates whether PEEK can perform effectively as a substitute material in fixed orthodontic appliances. After thorough examination of the research, the selected articles encompass the utilization of PEEK in esthetic orthodontic archwires, archwire sleeves, orthodontic brackets, and fixed lingual retainers. Due to the significant diversity among the studies regarding types of application, the designs and dimensions of the test specimens, methodology, and outcome assessments, it was not feasible to conduct a meta-analysis. However, based on the individual studies, the extent to which PEEK has been investigated as an alternative to fixed orthodontic appliances can be determined. 

Regarding orthodontic archwires, despite variations in the dimensions of PEEK orthodontic wires between Maekawa et al. [16] and Tada et al. [17], both investigations indicated that PEEK could withstand loads comparable to Ni-Ti wires. According to the previous studies, the optimal orthodontic force should be 0.27–1.1 N and for the central incisor, it was described as 0.45–0.59 N [25]. Therefore, Maekawa et al. suggested that a PEEK wire with “0.016 x 0.022 in^2^” dimensions can deliver around the above optimal force of 0.40–0.80 N. Therefore, PEEK shows promising prospects as a substitute material for orthodontic archwires. Tada et al. [17] performed a similar experiment to the previous study but with some modifications such as preparing the PEEK samples closer to the actual wire sizes and fixation of the wires into “0.022 × 0.028 in^2”^ bracket slots with different ligation methods. No significant difference was found in the level of permanent deformation and static friction between the PEEK and Ni-Ti wires. After a stress relaxation test, 70–80% of the initial load was still maintained for the two larger PEEK wire groups. Regarding the continuous optimum orthodontic force, Profit has suggested 0.5–1.5N [26]. According to this, the “0.019 × 0.025 in^2^” PEEK wire in SL ligation would be more effective than the “0.016 in” Ni-Ti control group and considered to be a suitable substitution. Additionally, over a longer duration of the above experiments, performing the test in an intra-oral environment and conducting a biofilm formation test should be further investigated.

Shirakawa et al. [18] proposed another alternative of using PEEK sleeves that are esthetically pleasing and also reduce the friction that occurs between the brackets and archwires. The friction coefficient between PEEK and wires is lower than that observed in traditional wire interaction. Moreover, using a PEEK tube not only increases the rate of leveling and alignment of the teeth but preserves the integrity of the bracket slot which can prevent an increase in torque play. 

To overcome ceramic bracket limitations, Wu et al. [19] attempted to implement PEEK as an orthodontic bracket substitute. After inspecting the samples under a laser profilometer, no significant surface roughness difference was found between the two materials. However, the PEEK group exhibited superior performance in terms of friction, potentially facilitating safer and more efficient tooth movement compared to ceramic brackets. Additionally, while ceramic’s abrasive wear generates debris that increases friction, PEEK’s adhesive wear transfers film onto steel counterparts, resulting in self-lubrication and reduced friction resistance. However, different types of wires, bonding strength, and biomechanical analysis should be further investigated. Overall, within the limitations of the study, PEEK demonstrates greater mechanical performance compared to ceramic material.

In the context of PEEK being utilized as a fixed lingual retainer, fundamental assessments primarily focused on its mechanical strength, bonding efficacy, and rigidity. The experiment conducted by Win et al. mainly focused on the mechanical strength and amount of deformation [23]. They determined that a hemi-elliptical, cross-sectional shape with a thickness of “0.039 in” (≈1.0 mm) and width of “0.059 in” (≈1.5 mm) would be the suitable dimensions for fabricating PEEK as fixed lingual retainer. It exhibited higher loading strength compared to clinically used SS retainer wires, such as a “0.028 × 0.008 in” flat metal wire and five-stranded SS wire, aiding in the prevention of bending and unwanted tooth movement. Furthermore, it not only demonstrated minimal permanent deformation but also maintained sufficient flexibility to accommodate physiologic tooth movement. Within the scope of the study, the author expressed that PEEK has the potential to serve as a suitable alternative option for orthodontic retainers. Further investigation including bonding strength tests and evaluations of the retainer’s performance in its actual curved form are yet to be conducted. Favorable outcomes regarding the bonding strength of PEEK were also observed from the experiment of Kadhum et al. [22]. In the article, PEEK was not only compared with other SS retainer wires but the appropriate surface treatment for better adhesion was also determined. Despite the various surface treatments, PEEK wires showed comparable performance to the other metal retainers except a “0.0195 in” dead-soft coaxial wire. Moreover, PEEK wires which were air-abraded and both air-abraded and treated with visio.link showed a greater pull-out force. However, air abrasion could weaken the strength of the PEEK material and cause rupture. Limitations of this study also included the straight piece of PEEK in a small, round, cross-sectional shape, using the protocol of cutting PEEK for crown and bridge work, manual finishing of the PEEK wires, and using bovine teeth instead of human teeth. Finally, the author suggested using a “0.031 in” (≈ 0.8 mm) PEEK wire with air abrasion alone and the application of a 4 mm composite bonding spot over the PEEK retainer.

After reviewing the above two articles, the results from Kadhum et al. appeared promising [22]. However, improving the failure force of the PEEK retainers would distinguish them among the competitors. Therefore, substituting the PEEK wire dimension suggested by Win et al. [23] with the bonding protocol recommended by Kadhum et al. [22] is expected to yield more satisfactory outcomes. 

To assess the long-term reliability of the PEEK retainer, Alabbadi et al. conducted a debonding procedure following six months of intra-oral clinical service stimulation [20]. Notably, the PEEK retainer successfully endured the aging procedure and proceeded to the debonding process. Following the procedure, the PEEK retainer showed a lower debonding force (45.73 ± 4.48 N) compared to the flat braided fixed retainer (86.81 ± 4.59 N). However, the debonding force of the PEEK retainer was significantly lower than that reported by Ruwiaee et al. where it reached 275 N [27]. Additionally, Kadhum et al. also reported a higher debonding force of 113.4 ± 18.21 N despite using bovine teeth and omitting an aging procedure [22]. This discrepancy may be attributed to differing surface treatments: the latter two studies involved surface treatment with 98% sulfuric acid for 60 s and air abrasion, respectively, [22,27] while the former applied a thin layer of bonding resin to the PEEK retainer [20]. Concerning ARI, PEEK scored three whereas the metal retainer scored either two or three. The results indicated a favorable outcome in minimizing enamel damage when debonding. Moreover, the PEEK retainer showed the least connector displacement among the test groups which lessened the risk of deformation by the low forces over a longer time, and avoid unwanted tooth movement. However, the author also proposed modifying the design into a less stiff wire form to balance physiologic tooth movement while preventing undesired tooth displacement. Hence, further research could consider employing the PEEK wire dimensions outlined by Win et al. [23]. Limitations of the study involve the utilization of premolar teeth rather than lower incisors, the shorter length of the retainer, and the omission of considerations for intra-oral environment factors.

Roser et al. also conducted two interrelated articles regarding a CAD/CAM fixed retainer including PEEK [21,24]. Unfortunately, all PEEK retainer samples dislodged during the aging process of 1,200,000 chewing cycles where a conventional twistflex retainer survived and was able to proceed to maximum load capacity test [21]. Nonetheless, the author chose not to apply any surface treatment to the PEEK retainer according to the manufacturer’s instruction, instead only sandblasting the model teeth. According to the previous studies [20,22,27], it could be confirmed that surface treatment for the PEEK retainer is imperative. Moreover, it was also discovered that integrating a retention pad for the PEEK retainer did not appear to offer any additional clinical advantages [20,21,22]. The limitation of tooth movement of the PEEK retainer was also investigated in another article [24]. Sadly, the PEEK retainer significantly restricted tooth mobility more than a conventional multistranded metal retainer. On the contrary, when reviewing the retainer dimensions, the utilization of larger dimensions, specifically a thickness of “0.047 in” (≈1.2 mm) and width of “0.138 in” (≈3.5 mm), may be the primary factor leading to a substantial degree of tooth movement restriction. Despite the use of CAD/CAM teeth models, Roser et als’ experiment demonstrated superiority in utilizing retainer samples in actual long, curved forms, closely resembling the clinical usage, in contrast to the straight retainer pieces [21,24]. The major limitation is that this review does not cover the biological and economical consideration of PEEK materials used in the fixed orthodontic appliances. Additionally, the applications of PEEK in removable appliances were also omitted. As PEEK is starting to be introduced into the orthodontic field, clinical trials are not yet available. Therefore, all the original articles conducted thus far have been limited to in vitro experimentation.

## 5. Conclusions

Based on the selected articles, the following conclusions can be drawn regarding PEEK-made orthodontic archwires, brackets, and fixed lingual retainers.

1. PEEK archwires exhibit the optimum orthodontic force, particularly the “0.019 × 0.025 in^2^” PEEK archwire in a “0.022 × 0.028 in” slot-lid ligation bracket which could provide a higher load than a “0.016 in” Ni-Ti wire. PEEK wire dimensions do not affect static friction. PEEK wires can maintain 70% to 80% of the initial load under a stress relaxation test for 24 h which was favorable for orthodontic treatment. Further investigation into load deflection, friction properties over a longer period, and intra-oral simulation are still needed. Moreover, PEEK tubes offer esthetic appeal and orthodontic efficiency.
2. PEEK brackets address ceramic bracket issues such as brittleness and bulkiness. They also demonstrate lower friction and adhesive wear characteristics, potentially replacing metal and ceramic materials in the future. As this study marks the initial exploration of PEEK brackets, more research is required to fully assess their potential in the clinical settings.
3. Research on PEEK fixed lingual retainers revealed that incorporating a retention pad into the PEEK fixed retainer did not enhance retention; rather, it impeded physiological tooth movement. Additionally, larger dimensions increase rigidity, raising the risk of debonding. Hence, wire-form PEEK retainers are preferred and a hemi-elliptical, cross-sectional shape with a thickness of “0.039 in” (≈1.0 mm) and a width of “0.059 in” (≈1.5 mm) should be used in further experiments. Moreover, surface treatment of PEEK retainers is necessary and a comparison between two methods, 98% sulfuric acid for 60s and air abrasion, should be conducted.

## Figures and Tables

**Table 1:** Characteristics of each study included in current systematic review.

No.	Author, Year	Application	Comparing Materials	Applied Tests	Conclusion
1.	Maekawa et al., 2015 [16]	Orthodontic wires	PEEK “0.039 × 0.039 in^2^” (≈1.0 × 1.0 mm^2^) Stainless steel (SS), Cobalt-chromium (Co-Cr), Titanium-molybdenum (Ti-Mo), Nickel-titanium (Ni-Ti) “0.016 × 0.022 in^2”^ (≈0.40 × 0.55 mm^2^)	Three-point bending test	PEEK showed highest bending strength and delivered similar force to Ni-Ti wire which was smaller in dimension.
2.	Tada et al., 2017 [17]	Orthodontic wires	PEEK (“0.016 in”, “0.016 × 0.022 in^2^”, “0.019 × 0.025 in^2^”) Ni-Ti (“0.016 in”)	Three-point bending test under three conditions (No ligation (NL), elastomeric ligation (EL), and slot lid ligation (SL)) Stress relaxation test Static friction test (EL only)	Use of “0.016 in” PEEK wire should be avoided. “0.019 × 0.025 in^2^” PEEK wire with the use of SL was suggested as an alternative to Ni-Ti wire.
3.	Shirakawa et al., 2018 [18]	Orthodontic wire sleeves	SS, Co-Cr, and Ni-Ti with and without PEEK tube (“0.018 in” and “0.017 × 0.025 in^2^”)	Friction test and surface roughness of a bracket slot (SS, Co-Cr, and Ni-Ti with and without PEEK tube)	Significantly lower friction values were recorded for all wires covered by the PEEK tube, except for “0.016 in” Ni-Ti where there was no significant difference. Alterations on the brackets were undetected when covered by the PEEK tube.
4.	Wu et al., 2024 [19]	Orthodontic bracket	PEEK, Ceramic	Surface roughness and morphology Coefficient of friction (COF)	PEEK can maintain a smooth surface similar to the ceramic brackets. PEEK also exhibited better mechanical properties and lower friction than ceramic.
5.	Alabbadi el al., 2023 [20]	Orthodontic fixed lingual retainer	PEEK, flat braided wire–Bond A. Braid	Aging procedure, failure bonding force test, measurement of connector retainer displacement, and adhesive remnant index (ARI)	A braided rectangular wire may be preferred due to its adequate debonding force, lower ARI, and greater permission of tooth movement compared to PEEK retainer.
6.	Roser et al., 2023 [21]	Orthodontic fixed lingual retainer	PEEK, stainless steel twistflex retainer	Aging followed by load capacity test	PEEK retainer exhibited the highest failure rate during aging and showed significantly lower strength compared to twistflex retainer. These findings indicate the limitations of long-term reliability.
7.	Kadhum et al., 2021 [22]	Orthodontic fixed lingual retainer	PEEK wire “0.031 in” (≈0.8 mm) with three surface preparations (no surface treatment, air abrasion, air abrasion followed by conditioning with a thin layer of visio.link) “0.0195 in” dead-soft coaxial wire, “0.010 × 0.028 in^2^” three strands stainless steel braided retainer wire, “0.010 × 0.028 in^2^” solid flat titanium dead-soft wire	Debonding from acrylic blocks, Debonding from bovine teeth, Pull-out test	Pre-treatment of PEEK wire with air abrasion alone showed comparable outcomes to the metallic retainer.
8.	Win et al., 2023 [23]	Orthodontic fixed lingual retainer	PEEK, stainless steel wire “0.036 in” (≈0.9 mm)	Three-point bending test via finite element analysis and laboratory test	Hemi-elliptical PEEK retainer with a thickness of “0.039 in” (≈1.0 mm) and width of “0.059 in” (≈1.5 mm) was mentioned as an optimal dimension to be used a fixed lingual retainer.
9.	Roser et al., 2024 [24]	Orthodontic fixed lingual retainer	PEEK “0.047 × 0.138 in^2^” (≈1.2 × 3.5 mm^2^), stainless steel twistflex retainer “0.022 in” (≈0.55 mm)	Horizontal and vertical tooth mobility test	PEEK retainer notably limits tooth mobility compared to multistranded retainers. Further experiments on modification of the PEEK retainer designs are needed. Selection of appropriate retainer types is required, particularly for patients with previous or high-risk periodontal damage.

**Table 2:** Risk of bias assessment using Quin Tool.

No.	Study	Signaling Questions												Summary
		I	II	III	IV	V	VI	VII	VIII	IX	X	XI	XII	
1	Maekawa et al., 2015 [16]	1	0	2	2	2	0	2	2	0	0	1	2	58.33% (M)
2	Tada et al., 2017 [17]	2	0	2	2	2	0	2	2	0	0	2	2	66.67% (M)
3	Shirakawa et al., 2018 [18]	2	0	2	2	2	0	2	2	0	0	2	2	66.67% (M)
4	Wu et al., 2024 [19]	2	0	2	2	2	0	2	2	0	0	2	2	66.67% (M)
5	Alabbadi et al., 2023 [20]	2	2	2	2	2	0	2	2	0	0	2	2	75.00% (L)
6	Roser et al., 2023 [21]	2	0	1	2	2	1	2	2	1	0	2	2	70.83% (L)
7	Kadhum et al., 2021 [22]	2	0	2	2	2	0	2	2	0	0	2	2	66.67% (M)
8	Win et al., 2023 [23]	2	0	2	2	2	0	2	2	0	0	2	2	66.67% (M)
9	Roser et al., 2024 [24]	2	0	2	2	2	0	2	2	0	0	2	2	66.67% (M)

I = clearly stated aims/objectives, II = detailed explanation of sample size calculation, III = detailed explanation of sampling technique, IV = detail of comparison group, V = detail explanation of methodology, VI = operator detail, VII = randomization, VIII = method of measurement of outcome, IX = outcome assessor details, X = blinding, XI = statistical analysis, XII = presentation of results. Adequately specified = 2 points, inadequately specified = 1 point, not specified = 0, not applicable = NA, L = low risk, M = medium risk, H = high risk.

## Data Availability

Data are contained within the article.
